# Bending and Vibration Analysis of Flexoelectric Beam Structure on Linear Elastic Substrates

**DOI:** 10.3390/mi13060915

**Published:** 2022-06-09

**Authors:** Maomao Zhang, Zhidong Zhou

**Affiliations:** 1 Fujian Provincial Key Laboratory of Advanced Materials, College of Materials, Xiamen University, Xiamen 361005, China; 18138803875@163.com; 2 Xiamen Key Laboratory of Electronic Ceramic Materials and Devices, College of Materials, Xiamen University, Xiamen 361005, China

**Keywords:** flexoelectric effect, linear elastic substrate, induced electric potential, magnetic field, natural frequency

## Abstract

With the development of micro-nanotechnology, smart electronic devices are being updated and developed, and more and more flexoelectric sensors, actuators, and energy harvesters attached to elastic substrates have attracted a surge of interest due to unique features at the nano-scale. In this paper, the static bending behavior and vibration characteristics of a flexoelectric beam structure based on a linear elastic substrate under a magnetic field environment are investigated. Based on the electrical Gibbs free energy density, the governing equations and boundary conditions of structures are derived by using the Euler–Bernoulli beam theory and the Hamilton’s variational principle. The expressions of the deflection and the induced electric potential of the beam structure are expressed analytically. The natural frequency of the beam under the open-circuit electrical conditions with surface electrodes (OCI) are obtained after further extending the solution. The results show that the flexoelectric effect, the linear elastic substrate, and the magnetic field have significant effects on the static bending and vibration behaviors of the flexoelectric beam which are beneficial for designing and developing flexoelectric devices with elastic substrates.

## 1. Introduction

With the rapid development of modern science and technology, there is a tendency toward ultra-precision and miniaturization in the smart material and smart structure research. There is a large market for smart devices at the nano-scale, when the flexoelectric effect, which is neglected at the macro-scale, plays an increasingly vital role due to its high electromechanical coupling. The flexoelectric effect is a kind of electromechanical coupling caused by strain gradients or non-uniform deformations [1,2,3,4], and this electromechanical phenomenon is size-dependent at the nano-scale [5]. Therefore, it is essential to understand and analyze the flexoelectric effect in nanoscale materials and structures.

Researchers have carried out many studies on the size-dependent static and vibration behaviors in micro-nanostructures considering the flexoelectric effect. Zhang et al. [5] established a Timoshenko dielectric beam model considering the direct flexoelectric effect and found that the deflection of cantilever and simply supported beams decreased with increasing beam thickness. Their results showed that flexoelectricity plays a major role in the electromechanical coupling response of piezoelectric beams when the beam thickness is at the nano-scale. Liang et al. [6,7] resolved and discussed the role of flexoelectric and surface effects in cantilever beam structures. Their results showed that surface and flexoelectric effects can reduce the bending deformation of the structure. Zhou et al. [8] investigated the flexoelectric effects in piezoelectric nanobeams with three different electrical boundary conditions based on the classical Euler–Bernoulli beam model. They gave the analytical expression of the induced electric potential of flexoelectric beams. Sladek et al. [9] analyzed curved nanoscale Timoshenko beams with the flexoelectric effect. The deflection, rotation, and induced electric intensity have been presented for various flexoelectric coefficients and the beam curvature. Park et al. [10] designed and analyzed a structural model based on piezoelectric polymer (PVDF) film with strain gradient response, and further extended the application of dielectric materials to flexoelectric sensors. Malikan and Eremeyev [11] modeled the dynamics of a visco-piezo-flexoelectric nanobeam considering a converse flexoelectric effect. Their results showed that the viscoelastic coupling will have an influence on the flexoelectricity property of the material. Yang and Zu [12] investigated the flexoelectric effect on the natural frequency of the conventional cantilever beam harvesting structure with an end mass block. Yan [13] elaborated the flexoelectric effect in composite flat plate harvesters by the weighted residual method based on the Kirchhoff plate theory. Liang et al. [14] studied the buckling and vibration of flexoelectric nanofilms under the mechanical loading. Chang [15,16] used the differential quadrature method and the finite element method to study the longitudinal vibration of nanobeams with variable cross-sections, respectively. Lin et al. [17] investigated the effects of end mass blocks and beam dimensions on the natural frequency and the effective frequency shift of a flexoelectric beam. Therefore, the study of flexoelectric structure properties is of directional guidance for the development and application of nanoresonators and nanosensors, etc.

Recently, there have been some research reports on nanostructures considering flexible substrates and magnetic fields. Hong [18] examined the static bending and free vibration of piezoelectric functionally graded plates on a two-parameter elastic foundation. Baradaran et al. [19] studied the surface effect on the static bending of nanowires on an elastic foundation. Ebrahimi and Barati [20] evaluated the buckling of flexoelectric nanobeams with an elastic foundation based on non-local and surface elasticity theories. They found that the nanostructures could tolerate higher buckling loads due to the flexoelectric and surface effects at the nano-scale. Yinusa et al. [21] analyzed the transverse and longitudinal vibrations and stability of the carbon nanotube in a magnetic environment. They determined that the magnetic term has a 20% attenuation or damping effect on the system vibration. Based on the variation method and the principle of minimum potential energy, Gobadi et al. [22] studied the thermo-electro-magnetic mechanical behavior of flexoelectric nanoplates, in which the analytical solutions have been presented. Akgoz and Civalek [23] analyzed the size-dependent stability of single-walled carbon nanotubes surrounded by a two-parameter elastic substrate. They found that increasing the Winkler and Pasternak parameters can increase the buckling load of carbon nanotubes. Jalaei et al. [24] studied the transient response of viscoelastic functionally graded nanobeams under dynamic loads and magnetic fields. The results showed that the oscillation amplitude decreases while the number of periods of nanobeams increases by increasing the magnetic field and the length scale parameter. Barati [25] analyzed the vibration characteristics of flexoelectric beams attached to a nonlinear foundation under the short-circuit electrical condition based on the surface elasticity and non-local elasticity theories. Recently, Xu et al. [26] established a rectangular piezoelectric cantilever beam energy harvester with a copper substrate. Employing the finite element method, the influence of the copper substrate size on the output performance of piezoelectric harvesters was analyzed, and the optimal size of the substrate was obtained to achieve the maximum voltage output at a low frequency. The substrates and external fields have significant effects on the electromechanical properties of flexoelectric or piezoelectric actuators, sensors or harvesters. It is not clear how the coupling of substrate parameters and magnetic fields acts on the electromechanical responses of sensors or harvesters. However, to our knowledge, none of the previous studies mentioned how the linear elastic substrate and magnetic fields affect the static bending and vibration behaviors of flexoelectric sensors under the open-circuit electrical condition. The induced electric potential and the natural frequency under the OCI condition are very important performances of the flexoelectric sensors or energy harvesters.

The purpose of the present paper was to study the bending behavior and vibration properties of a flexoelectric cantilever beam attached to a linear elastic substrate under the OCI condition. Based on the electrical Gibbs free energy density and the Hamilton’s variational principle, the dynamic governing equations and the corresponding general boundary conditions were derived. Then, the characteristic equations of the natural frequency and the static electromechanical responses were further obtained. The bending behavior, vibration response, and the effects of linear elastic parameters and magnetic field on structural performance are discussed in detail.

## 2. Basic Theory of Flexoelectric Materials

Based on the electrical Gibbs free energy density function, according to the traditional piezoelectric theory, the basic theoretical model of the flexoelectric material is constructed with the interaction between the electric field and the strain gradient. Thereby, electrical Gibbs free energy density function U of the material can be written as:

(1) $$U=-\frac{1}{2}a_{ij}E_{i}E_{j}+\frac{1}{2}c_{ijkl}\varepsilon_{ij}\varepsilon_{kl}-e_{ijk}E_{i}\varepsilon_{jk}+f_{ijkl}E_{i}\varepsilon_{jk,l}+d_{ijkl}E_{i,j}\varepsilon_{kl}$$

where $a_{ij},\;c_{ijkl},\;e_{ijk},\;f_{ijkl}$, and $d_{ijkl}$ are the material property parameters, respectively. $a_{ij}$ denotes the dielectric coefficient, $c_{ijkl}$ denotes the modulus of elasticity, $e_{ijk}$ is the piezoelectric coefficient, $f_{ijkl}$ denotes the positive flexoelectric coefficient, and $d_{ijkl}$ denotes the inverse flexoelectric coefficient. $E_{i}$ is the electric field, $E_{i,j}$ is the electric field gradient, $\varepsilon_{ij}$ denotes the strain, and $\varepsilon_{jk,l}$ denotes the strain gradient. Sharma et al. [27] investigated the flexoelectric effect equivalent to piezoelectricity in bending film and defined $\mu_{ijkl}=d_{ijkl}-f_{ijkl}$ as the effective flexoelectric coefficient. Considering the effective flexoelectric coefficient, Equation (1) can be rewritten as:

(2) $$U=-\frac{1}{2}a_{ij}E_{i}E_{j}+\frac{1}{2}c_{ijkl}\varepsilon_{ij}\varepsilon_{kl}-e_{ijk}E_{i}\varepsilon_{jk}-\mu_{ijkl}E_{i}\varepsilon_{jk,l}$$

Under the assumption of linear deformation, the expression between the strain and strain gradient and its displacement $u_{i}$ is:

(3) $$\varepsilon_{ij}=\frac{1}{2}\left(u_{i,j}+u_{j,i}\right)$$

(4) $$\varepsilon_{ij,k}=\frac{1}{2}\left(u_{i,jk}+u_{j,ik}\right)$$

Correspondingly, the constitutive equations for flexoelectric materials can be further obtained under linear small deformation conditions:

(5) $$\sigma_{ij}=\frac{\partial U}{\partial \varepsilon_{ij}}=c_{ijkl}\varepsilon_{kl}-e_{ijk}E_{k}$$

(6) $$\sigma_{ijk}=\frac{\partial U}{\partial \varepsilon_{ij,k}}=-\mu_{ijkl}E_{l}$$

(7) $$D_{i}=-\frac{\partial U}{\partial E_{i}}=a_{ij}E_{j}+e_{ijk}\varepsilon_{jk}+\mu_{ijkl}\varepsilon_{jk,l}$$

where, $\sigma_{ij},\;\sigma_{ijk},\;D_{i}$ are the Cauchy stress tensor, the higher-order stress tensor, and electric displacement vector, respectively. Substituting Equations (5)–(7) into Equation (2), an alternative expression for the electrical Gibbs free energy density function is obtained:

(8) $$U=\frac{1}{2}\sigma_{ij}\varepsilon_{ij}+\frac{1}{2}\sigma_{ijk}\varepsilon_{ij,k}-\frac{1}{2}D_{i}E_{i}$$

## 3. Analysis Model of Flexoelectric Beams Based on Linear Elastic Substrates

**Figure 1:**
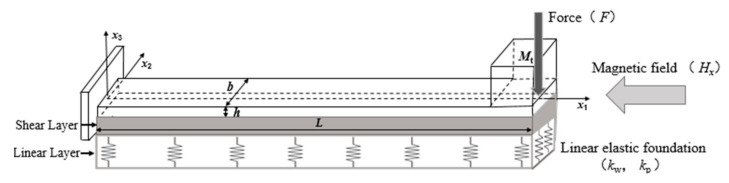
The model of a flexoelectric cantilever beam based on a linear elastic substrate.

The present research object is the flexoelectric cantilever beam structure with an end mass based on linear elastic substrates. The cantilever beam model is shown in Figure 1. The length, width, and thickness of the cantilever beam are L, b, and h, respectively. The mass of the end mass block is $M_{t}$, where the end mass block is set as a cube and the side length is d=b. The force *F* is applied at the end of the beam. The top and bottom surfaces of the beam are covered with electrodes, in which the thickness and stiffness of electrode layers would be neglected. This cantilever beam structure is connected to a linear elastic substrate and subjected to an in-plane axial magnetic field $H_{x}$. The linear elastic substrate can be simulated by a two-parameter linear elastic foundation model consisting of linear and shear layers. Here, $k_{p}$ is the Pasternak constant, which describes the shear effect, and $k_{w}$ is the Winkler constant, which describes the tensile effect.

The Hamilton’s variational expression for a flexoelectric cantilever beam is [6]:

(9) $$\delta \int_{0}^{T}\left(K-G+W\right)dt=0$$

where K, G, and W are the total kinetic energy of the system, the total electrical Gibbs free energy, and the work done by the external loads, respectively. Equation (9) can be written as:

(10) $$\left\{\begin{array}{c} K=\int_{V}^{}\frac{1}{2}\rho {\left|{\dot{w}}^{m}\right|}^{2}dV+\left[\frac{1}{2}M_{t}{\left|\frac{\partial \left(w+yw^{\prime }\right)}{\partial t}\right|}^{2}+\frac{1}{2}I_{t}{\left|\frac{\partial {\dot{w}}^{m}}{\partial x_{1}}\right|}^{2}\right]{|}_{x_{1}=L}\\ G=\int_{V}^{}UdV\\ W=\oint_{s}^{}\varpi \phi dA_{0}+\frac{1}{2}Fw\left|\begin{array}{c} L\\ 0 \end{array}\right.+W_{e} \end{array}\right.$$

where ρ is the flexoelectric material density, ${\dot{w}}^{m}$ is the primary derivative of the absolute displacement of the flexoelectric beam with respect to time, where $w^{m}\left(x_{1},t\right)=w\left(x_{1},t\right)$. $w\left(x_{1},t\right)$ denotes the transverse displacement of the neutral layer of the beam along the $x_{3}$ direction, which is the deflection of the beam. $I_{t}$ is the moment of inertia corresponding to the end mass block. $\varpi \left(x_{1},\;t\right)$ denotes the free charge density of the electrodes on the top and bottom surfaces of the beam. $\phi \left(x_{1},\;t\right)$ is the electric potential on the surfaces generated by the bending of the cantilever beam. V and $A_{0}$ are the volume and top and bottom surface areas of the beam, respectively. $W_{e}$ is the work performed on the system by the external conditions (the substrate and magnetic field). For a planar beam deformation, combined with the Maxwell relation, the form of the Lorentz force generated under the action of the magnetic field can be expressed as: $f_{Lz}=\xi AH_{x}^{2}\frac{d^{2}w}{dx^{2}}$ [28], where $f_{Lz}$ is the Lorentz force, ξ is magnetic parliamentary, and A is the cross-sectional area of the beam. Combined with the two-parameter foundation model, the work variation of the external fields can be written as: $\delta W_{e}=\int_{0}^{L}\left(-k_{w}w+k_{p}\frac{\partial^{2}w}{\partial x_{1}^{2}}+\xi AH_{x}^{2}\frac{\partial^{2}w}{\partial x_{1}^{2}}\right)\delta wdx_{1}$ [20,25,28].

According to the Euler–Bernoulli theory, the displacement expression of the flexoelectric beam is:

(11) $$u_{1}=-x_{3}\frac{dw}{dx_{1}},\;u_{2}=0,w=w\left(x_{1},\;t\right)$$

where $u_{1},\;u_{2}$ are the displacements along $x_{1}\;$ and $x_{2}$ directions, respectively, and w is the deflection of the beam structure as bending. The expression of strain and strain gradient of the flexoelectric cantilever beam is:

(12) $$\varepsilon_{11}=-x_{3}\frac{d^{2}w}{dx_{1}^{2}},\;\varepsilon_{11,3}=-\frac{d^{2}w}{dx_{1}^{2}},\;\varepsilon_{11,1}=-x_{3}\frac{d^{3}w}{dx_{1}^{3}}$$

Under the condition of linear small deformation, the constitutive equations of the flexoelectric material can be further obtained:

(13) $$\sigma_{11}=c_{11}\varepsilon_{11}-e_{311}E_{3}$$

(14) $$\sigma_{113}=-\mu_{3113}E_{3}$$

(15) $$D_{3}=a_{33}E_{3}+e_{311}\varepsilon_{11}+\mu_{3113}\varepsilon_{11,3}$$

The electric field $E_{3}$ inside the cantilever beam can also be expressed as a negative gradient of the internal electric potential $\Phi_{0}\left(x_{1},\;x_{3},\;t\right)$ along the thickness direction:

(16) $$E_{3}=-\frac{\partial \Phi_{0}}{\partial x_{3}}$$

No free charge exists in flexoelectric cantilever beams, and thus, the electric displacement should satisfy the Gauss’s law, i.e., $D_{3,3}=0$. After substituting Equation (15) into Gauss’s law, combined with Equation (16), we obtained:

(17) $$\frac{\partial^{2}\Phi_{0}}{\partial x_{3}^{2}}=-\frac{e_{311}}{a_{33}}\frac{d^{2}w}{dx_{1}^{2}}$$

Under the OCI condition, it is assumed that the electric potential on the top surface of the cantilever beam is $\Phi_{0}\left(x_{1},\frac{h}{2},\;t\right)=0$ and the electric potential on the bottom surface is $\Phi_{0}\left(x_{1},-\frac{h}{2},\;t\right)=\phi \left(x_{1},\;t\right)$. Then the electric potential difference between the top and bottom surfaces of the beam is $\phi \left(x_{1},\;t\right)$. Solving Equation (17) and combining with the electrical boundary conditions, the expression for the internal electric potential of the flexoelectric cantilever beam can be obtained:

(18) $$\Phi_{0}\left(x_{1},x_{3},\;t\right)=-\frac{e_{311}}{2a_{33}}\frac{d^{2}w}{dx_{1}^{2}}x_{3}^{2}-\frac{\phi \left(x_{1},\;t\right)}{h}x_{3}+C\left(x_{1,},\;t\right)$$

where $C\left(x_{1},\;t\right)$ is the potential value of the neutral axis of the beam with respect to $x_{1}$. By the above equation, the electric field $E_{3}$, stress $\sigma_{11}$, higher order stress $\sigma_{113}$, and electric displacement $D_{3}$ can be obtained:

(19) $$E_{3}=-\frac{e_{311}}{a_{33}}\varepsilon_{11}+\frac{\phi \left(x_{1},\;t\right)}{h}$$

(20) $$\sigma_{11}=(c_{11}+\frac{e_{311}^{2}}{a_{33}})\varepsilon_{11}-e_{311}\frac{\phi \left(x_{1},\;t\right)}{h}$$

(21) $$\sigma_{113}=\mu_{3113}\frac{e_{311}}{a_{33}}\varepsilon_{11}-\mu_{3113}\frac{\phi \left(x_{1},\;t\right)}{h}$$

(22) $$D_{3}=\mu_{3113}\varepsilon_{11,3}+a_{33}\frac{\phi \left(x_{1},\;t\right)}{h}$$

Substituting Equations (19)–(22) into the Equation (8), we can obtain the expansion of the electrical Gibbs free energy density function U of the cantilever beam structure:

(23) $$U=\frac{1}{2}(c_{11}+\frac{e_{311}^{2}}{a_{33}})\varepsilon_{11}^{2}+\frac{\mu_{3113}e_{311}}{a_{33}}\varepsilon_{11}\varepsilon_{11,3}-\frac{\mu_{3113}\phi \left(x_{1},\;t\right)}{h}\varepsilon_{11,3}-\frac{1}{2}a_{33}\frac{\phi^{2}\left(x_{1},\;t\right)}{h^{2}}$$

U is a functional expression related to time *t* in the vibration. Therefore, the electrical Gibbs free energy density function could be expanded by the generalized variational method, i.e.,

(24) $$\begin{array}{ll} \delta \int_{0}^{T}dt\int_{V}^{}UdV & =\int_{0}^{T}\int_{0}^{L}\left[\left(G_{P}\frac{\partial^{4}w}{\partial x_{1}^{4}}+\mu_{3113}b\frac{\partial^{2}\phi }{\partial x_{1}^{2}}\right)\delta w+\left(\mu_{3113}b\frac{\partial^{2}w}{\partial x_{1}^{2}}-a_{33}\frac{\phi b}{h}\right)\delta \phi \right]dx_{1}dt\\ & +\int_{0}^{T}\left(G_{P}\frac{\partial^{2}w}{\partial x_{1}^{2}}+\mu_{3113}b\phi \right)\delta \left(\frac{\partial w}{\partial x_{1}}\right){|}_{x_{1}=L}dt-\int_{0}^{T}G_{P}\frac{\partial^{3}w}{\partial x_{1}^{3}}\delta w{|}_{x_{1}=L}dt \end{array}$$

where $G_{P}=\frac{bh^{3}}{12}(c_{11}+\frac{e_{311}^{2}}{a_{33}})$ is the effective bending rigidity of the piezoelectric nanobeam.

By using the parallel axis theorem, the expression for the moment of inertia of the end mass block can be obtained as:

(25) $$I_{t}=\frac{1}{6}M_{t}d^{2}+M_{t}{\left(\frac{d+h}{2}\right)}^{2}$$

Then, by substituting Equations (24) and (25) into Equation (9), the generalized Hamilton’s variational equation of the flexoelectric cantilever beam structure is obtained:

(26) $$\begin{array}{l} \int_{0}^{T}dt\int_{V}^{}\rho \left(\ddot{w}\right)\delta wdV\\ \;\;\;\;\;\;\;+\int_{0}^{T}dt\int_{0}^{L}[\left(G_{p}\frac{\partial^{4}w}{\partial x_{1}^{4}}+\mu_{3113}b\frac{\partial^{2}\phi }{\partial x_{1}^{2}}+k_{w}w-k_{p}\frac{\partial^{2}w}{\partial x_{1}^{2}}-\xi AH_{x}^{2}\frac{\partial^{2}w}{\partial x_{1}^{2}}\right)\delta w\\ \;\;\;\;\;\;\;-\left(\mu_{3113}b\frac{\partial^{2}w}{\partial x_{1}^{2}}+a_{33}\frac{\phi b}{h}\right)\delta \phi ]dx_{1}+\int_{0}^{T}\left(G_{p}\frac{\partial^{2}w}{\partial x_{1}^{2}}+\mu_{3113}b\phi +I_{t}\frac{\partial^{3}w}{\partial x_{1}\partial t^{2}}\right)\delta \left(\frac{\partial w}{\partial x_{1}}\right){|}_{x_{1}=L}dt\\ \;\;\;\;\;\;\;-\int_{0}^{T}\left(G_{p}\frac{\partial^{3}w}{\partial x_{1}^{3}}+F-M_{t}\frac{\partial^{2}w}{\partial t^{2}}\right)\delta w{|}_{x_{1}=L}dt+\int_{0}^{T}dt\int_{S}^{}\varpi \delta \phi dA_{0}=0 \end{array}$$

where $\ddot{w}$ is the second derivative of the deflection w with respect to time, $\delta \left(x_{1}\right)$ is the Diracdelta function. The following variational expansion is applied in the further derivation of Equation (26):

(27) $$\left\{\begin{array}{c} \delta \int_{0}^{T}\frac{1}{2}M_{t}{\left(\frac{\partial w}{\partial t}\right)}^{2}dt{|}_{x_{1}=L}=-M_{t}\int_{0}^{T}\frac{\partial^{2}w}{\partial t^{2}}\delta wdt{|}_{x_{1}=L}\\ \delta \int_{0}^{T}\frac{1}{2}I_{t}{\left|\frac{\partial {\dot{w}}^{m}}{\partial x_{1}}\right|}^{2}dt{|}_{x_{1}=L}=-\int_{0}^{T}I_{t}\frac{\partial^{3}w}{\partial x_{1}\partial t^{2}}\delta \left(\frac{\partial w}{\partial x_{1}}\right)dt{|}_{x_{1}=L} \end{array}\right.$$

### 3.1. The Bending Response of Flexoelectric Beams Attached to Linear Elastic Substrates

The top and bottom surfaces of a flexoelectric cantilever beam are covered with electrodes, thus the top and bottom surfaces are electrical equipotential bodies and the electric potential difference $\phi \left(t\right)$ is a function independent of $x_{1}$. In the analysis of the beam structure statically, let *t* = 0 and $M_{t}\;$= 0. For any δw, Equation (26) is satisfied, in which the electromechanical coupling governing equation and boundary conditions of the flexoelectric cantilever beam under the OCI condition can be obtained:

(28) $$G_{P}\frac{d^{4}w}{dx_{1}^{4}}-\left(k_{p}+\xi AH_{x}^{2}\right)\frac{d^{2}w}{dx_{1}^{2}}+k_{w}w=0$$

(29) $$\left\{\begin{array}{c} w=\frac{dw}{dx_{1}}=0,\;\left(x_{1}=0\right)\\ G_{P}\frac{d^{2}w}{dx_{1}^{2}}+\mu_{3113}b\phi =0,\;G_{P}\frac{d^{3}w}{dx_{1}^{3}}+F=0\;\left(x_{1}=L\right) \end{array}\right.$$

Similarly, for Equation (26), δϕ can be chosen arbitrarily, and there is the following relation:

(30) $$\int_{0}^{L}(\varpi +\mu_{3113}\frac{d^{2}w}{dx_{1}^{2}}-a_{33}\frac{\phi }{h})dx_{1}=\int_{0}^{L}\left(\varpi -D_{3}\right)dx_{1}=0$$

Under OCI conditions, the charges on the bending surfaces of the flexoelectric beam are redistributed, however, the total surface free charge or the surface electric displacement of the flexoelectric beam is zero [8,29,30]. Thus, the electrical boundary condition expression is:

(31) $$\int_{0}^{L}\left(\mu_{3113}\frac{d^{2}w}{dx_{1}^{2}}-a_{33}\frac{\phi }{h}\right)dx_{1}=0\;\mathrm{or}\;\int_{0}^{L}-D_{3}dx_{1}=0$$

So, the electric potential could be obtained $\;\phi \left(t\right)=\frac{\mu_{3113}h}{a_{33}L}\frac{\partial w}{\partial x_{1}}{|}_{x_{1}=L}$ for the flexoelectric cantilever beam.

Therefore, the expressions of the deflection and induced electric potential as only force F (case I) are:

(32) $$w_{1}=\frac{3FLx_{1}^{2}-Fx_{1}^{3}}{6G_{P}}-\frac{\mu_{3113}{}^{2}bFLhx_{1}^{2}}{4G_{P}\left(\mu_{3113}^{2}bh+G_{P}a_{33}\right)}$$

(33) $$\phi_{1}=\frac{\mu_{3113}FLh}{2\left(\mu_{3113}^{2}bh+G_{P}a_{33}\right)}$$

The expressions of the deflection and induced electric potential as the force F and the magnetic field (case II) are:

(34) $$\begin{array}{cc} w_{2}=\frac{1}{2r^{3}G_{P}} & \left\{\frac{\left[\sin h\left(rx_{1}\right)+\cos h\left(rx_{1}\right)\right]\left[-F-r\mu_{3113}b\phi \right]}{\left[\sin h\left(rL\right)+\cos h\left(rL\right)\right]}+\frac{\left[\sin h\left(rx_{1}\right)-\cos h\left(rx_{1}\right)\right]\left[F-r\mu_{3113}b\phi \right]}{\left[\sin h\left(rL\right)-\cos h\left(rL\right)\right]}\right\}\\ & +\frac{\left[r^{2}\mu_{3113}b\phi \sin h\left(rL\right)-rF\cos h\left(rL\right)\right]x_{1}+F\sin h\left(rL\right)-r\mu_{3113}b\phi \cos h\left(rL\right)}{r^{3}G_{P}\left[\sin^{2}h\left(rL\right)-\cos^{2}h\left(rL\right)\right]} \end{array}$$

(35) $$\phi_{2}=\frac{h\mu_{3113}F\left[\sin^{2}h\left(rL\right)-\cos^{2}h\left(rL\right)+\cos h\left(rL\right)\right]}{r\left\{\mu_{3113}^{2}bh\sin h\left(rL\right)-a_{33}rLG_{P}\left[\sin^{2}h\left(rL\right)-\cos^{2}h\left(rL\right)\right]\right\}}$$

where $:\;r=\sqrt{\frac{\xi AH_{x}^{2}}{G_{P}}}$.

Based on a linear elastic substrate, the expressions of the deflection and induced electric potential as only force F (case III) are:

(36) $$\begin{array}{cc} w_{3}= & \left\{\frac{4\mu_{3113}^{2}b\alpha_{1}nhF\left(m_{1}\alpha_{1}u_{1}+t_{1}u_{2}\right)}{Z\left[2\mu_{3113}^{2}b\alpha_{1}h\left(\alpha_{1}nu_{1}+m_{1}u_{2}\right)-Za_{33}L\right]}-\frac{2Fm_{1}}{Z}\right\}\left[\sin h\left(\alpha_{1}x_{1}\right)-\frac{\alpha_{1}}{\alpha_{2}}\sin h\left(\alpha_{2}x_{1}\right)\right]\\ & +\left\{\frac{4\mu_{3113}^{2}b\alpha_{1}m_{1}hF\left(m_{1}\alpha_{1}u_{1}+t_{1}u_{2}\right)}{Z\left[2\mu_{3113}^{2}b\alpha_{1}h\left(\alpha_{1}nu_{1}+m_{1}u_{2}\right)-Za_{33}L\right]}-\frac{2Ft_{1}}{Z}\right\}\left[\cos h\left(\alpha_{2}x_{1}\right)-\cos h\left(\alpha_{1}x_{1}\right)\right] \end{array}$$

(37) $$\phi_{3}=\frac{h\mu_{3113}F\left(m_{1}\alpha_{1}u_{1}+t_{1}u_{2}\right)}{2\mu_{3113}^{2}b\alpha_{1}h\left(\alpha_{1}nu_{1}+m_{1}u_{2}\right)-Za_{33}L}$$

where:

$$s_{1}=\frac{k_{p}}{G_{P}},s_{2}=\frac{k_{w}}{G_{P}},\alpha_{1}=\sqrt{\frac{s_{1}+\sqrt{s_{1}^{2}-4s_{2}}}{2}},\alpha_{2}=\sqrt{\frac{s_{1}-\sqrt{s_{1}^{2}-4s_{2}}}{2}},$$

$$m_{1}=\alpha_{1}^{3}\cos h\left(\alpha_{1}L\right)-\alpha_{1}\alpha_{2}^{2}\cos h\left(\alpha_{2}L\right),n=\alpha_{1}^{3}\sin h\left(\alpha_{1}L\right)-\alpha_{2}^{3}\sin h\left(\alpha_{2}L\right),$$

$$t_{1}=\alpha_{1}^{3}\sin h\left(\alpha_{1}L\right)-\alpha_{1}^{2}\alpha_{2}\sin h\left(\alpha_{2}L\right),\;Z=2G_{P}\left(m_{1}^{2}-nt_{1}\right)$$

Based on a linear elastic substrate, the expressions of the deflection and induced electric potential acting on the force F and the magnetic field (case IV) are the same as in the case III, with the difference of the parameter $s_{1}$ which should be changed to $s_{1}=\frac{k_{p}+\xi AH_{x}^{2}}{G_{P}}$.

### 3.2. The Vibration Response of Flexoelectric Beams Attached to Linear Elastic Substrates

When the vibration characteristics of the beam structure are analyzed, *t* ≠ 0 and *F* = 0 have been set. Therefore, Equation (26) can be satisfied for any choice of δw, so that the dynamic governing equation of the flexoelectric cantilever beam system under the OCI condition can be obtained:

(38) $$G_{p}\frac{\partial^{4}w}{\partial x_{1}^{4}}+k_{w}w-\left(k_{p}+\xi AH_{x}^{2}\right)\frac{\partial^{2}w}{\partial x_{1}^{2}}+m\frac{\partial^{2}w}{\partial t^{2}}=0$$

where m=ρbh denotes the mass per unit length of the flexoelectric beam.

Correspondingly, the dynamic induced electric potential could be expressed as $\phi \left(t\right)=\frac{\mu_{3113}h}{a_{33}L}\frac{\partial w\left(t\right)}{\partial x_{1}}{|}_{x_{1}=L}$. From Equation (26), the dynamic mechanical boundary conditions of the flexoelectric beam based on the linear elastic substrate under the OCI condition can be obtained:

(39) $$\left\{\begin{array}{c} w\left(t,0\right)=0\\ \frac{\partial w\left(t\right)}{\partial x_{1}}{|}_{x_{1}=0}=0\\ \left[G_{p}\frac{\partial^{2}w\left(t\right)}{\partial x_{1}^{2}}+\frac{\mu_{3113}^{2}bh}{a_{33}L}\frac{\partial w\left(t\right)}{\partial x_{1}}+I_{t}\frac{\partial^{3}w\left(t\right)}{\partial x_{1}\partial t^{2}}\right]{|}_{x_{1}=L}=0\\ \left[G_{p}\frac{\partial^{3}w\left(t\right)}{\partial x_{1}^{3}}-M_{t}\frac{\partial^{2}w\left(t\right)}{\partial t^{2}}\right]{|}_{x_{1}=L}=0 \end{array}\right.$$

By the separated variables method, the characteristic equation for the natural frequency of the flexoelectric energy harvester based on a linear elastic substrate under the OCI condition can be solved. The solution of this equation can be set according to the form of Equation (38) as [31,32]:

(40) $$w\left(x_{1},t\right)=\emptyset \left(x_{1}\right)\eta \left(t\right)$$

(41) $$\left\{\begin{array}{c} \emptyset \left(x_{1}\right)=A_{1}sin\beta_{1}x_{1}+A_{2}cos\beta_{1}x_{1}+A_{3}sinh\beta_{2}x_{1}+A_{4}cosh\beta_{2}x_{1}\\ \eta \left(t\right)=A_{5}e^{\lambda it} \end{array}\right.$$

where $\emptyset \left(x_{1}\right)$ denotes the modal vibration pattern, $\beta_{1},\;\beta_{2}$ are the eigenvalues of the structural vibration, the five parameters $A_{1},\;\;A_{2}$, $A_{3}$,$\;A_{4}$, and $A_{5}$ are independent of t and $x_{1}$, $\eta \left(t\right)$ denotes the generalized coordinate, and i is the imaginary root. After substituting Equations (40) and (41) into Equations (38) and (39), the dynamic governing equation and the corresponding boundary conditions of the cantilever beam structure can be obtained:

(42) $$G_{p}\frac{d^{4}\emptyset \left(x_{1}\right)}{dx_{1}^{4}}-\left(k_{p}+\xi AH_{x}^{2}\right)\frac{d^{2}\emptyset \left(x_{1}\right)}{dx_{1}^{2}}+k_{w}\emptyset \left(x_{1}\right)-m\lambda^{2}\emptyset \left(x_{1}\right)=0$$

(43) $$\left\{\begin{array}{c} \emptyset \left(0\right)=0\\ \frac{d\emptyset \left(x_{1}\right)}{dx_{1}}{|}_{x_{1}=0}=0\\ \left[G_{p}\frac{d^{2}\emptyset \left(x_{1}\right)}{dx_{1}^{2}}+\left(\frac{\mu_{3113}^{2}bh}{a_{33}L}-I_{t}\lambda^{2}\right)\frac{d\emptyset \left(x_{1}\right)}{dx_{1}}\right]{|}_{x_{1}=L}\\ \left[G_{p}\frac{d^{3}\emptyset \left(x_{1}\right)}{dx_{1}^{3}}+\lambda^{2}M_{t}\emptyset \left(x_{1}\right)\right]{|}_{x_{1}=L}=0 \end{array}\right.=0$$

The circular frequency expression of the natural vibration of the system can be obtained from Equation (42): $\lambda =\sqrt{\frac{k_{w}-\left(k_{p}+\xi AH_{x}^{2}\right)\beta^{2}+G_{p}\beta^{4}}{m}}$. Then, the following relation can be obtained after substituting Equation (41) into Equation (43):

(44) $$\left\{\begin{array}{c} B_{1}A_{1}+B_{2}A_{2}=0\\ B_{3}A_{1}+B_{4}A_{2}=0 \end{array}\right.$$

In which:

(45) $$\left\{\begin{array}{c} B_{1}=G_{p}\left(-\beta_{1}^{2}\sin\beta_{1}L-\beta_{1}\beta_{2}\sin h\beta_{2}L\right)+\left(\frac{\mu_{3113}^{2}bh}{a_{33}L}-I_{t}\lambda^{2}\right)\left(\beta_{1}\cos\beta_{1}L-\beta_{1}\cos h\beta_{2}L\right)\\ B_{2}=G_{p}\left(-\beta_{1}^{2}\cos\beta_{1}L-\beta_{2}^{2}\cos h\beta_{2}L\right)+\left(\frac{\mu_{3113}^{2}bh}{a_{33}L}-I_{t}\lambda^{2}\right)\left(-\beta_{1}\sin\beta_{1}L-\beta_{2}\sin h\beta_{2}L\right)\\ B_{3}=G_{p}\left(-\beta_{1}^{3}\cos\beta_{1}L-\beta_{1}\beta_{2}^{2}\cos h\beta_{2}L\right)+\lambda^{2}M_{t}\left(\sin\beta_{1}L-\frac{\beta_{1}}{\beta_{2}}\sin h\beta_{2}L\right)\\ B_{4}=G_{p}\left(\beta_{1}^{3}\sin\beta_{1}L-\beta_{2}^{3}\sin h\beta_{2}L\right)+\lambda^{2}M_{t}\left(\cos\beta_{1}L-\cos h\beta_{2}L\right) \end{array}\right.$$

$$r_{1}=\frac{k_{p}+\xi AH_{x}^{2}}{G_{p}},\;r_{2}=\frac{k_{w}-m\lambda^{2}}{G_{p}},$$

$$\beta_{1}={\left[\frac{{\left(r_{1}^{2}-4r_{2}\right)}^{1/2}-r_{1}}{2}\right]}^{1/2},\;\beta_{2}={\left[\frac{r_{1}+{\left(r_{1}^{2}-4r_{2}\right)}^{1/2}}{2}\right]}^{1/2}$$

To ensure that the system of homogeneous Equation (44) has non-zero solutions, the coefficient determinant corresponding to the equations must be zero. By solving and simplifying the matrix determinant, the characteristic equation for the natural frequency of this flexoelectric energy harvester under the OCI condition can be obtained as:

(46) $$\begin{array}{ll} G_{p}^{2}[-\beta_{1}^{5}\left(S_{1}^{2}+C_{1}^{2}\right) & +\beta_{1}^{2}\beta_{2}^{2}\left(\beta_{2}S_{1}S_{2}-\beta_{1}C_{1}C_{2}\right)-\beta_{1}^{3}\beta_{2}\left(\beta_{1}S_{1}S_{2}+\beta_{2}C_{1}C_{2}\right)+\beta_{1}\beta_{2}^{4}\left(S_{2}^{2}-C_{2}^{2}\right)]\\ & +G_{p}\lambda^{2}M_{t}\left[\beta_{1}^{2}\left(S_{1}C_{2}-\frac{\beta_{1}}{\beta_{2}}S_{2}C_{1}\right)+\beta_{2}\left(\beta_{2}S_{1}C_{2}-\beta_{1}S_{2}C_{1}\right)\right]\\ & -G_{p}\left(\frac{\mu_{3113}^{2}bh}{a_{33}L}-I_{t}\lambda^{2}\right)\left[\beta_{1}\beta_{2}^{2}\left(\beta_{1}S_{1}C_{2}+\beta_{2}S_{2}C_{1}\right)+\beta_{1}^{3}\left(\beta_{2}S_{2}C_{1}+\beta_{1}S_{1}C_{2}\right)\right]\\ & -\lambda^{2}M_{t}\left(\frac{\mu_{3113}^{2}bh}{a_{33}L}-I_{t}\lambda^{2}\right)[-\beta_{1}\left(S_{1}^{2}+C_{1}^{2}\right)+\beta_{1}\left(\frac{\beta_{1}}{\beta_{2}}S_{1}S_{2}+C_{1}C_{2}\right)+\left(\beta_{1}C_{1}C_{2}-\beta_{2}S_{1}S_{2}\right)\\ & +\beta_{1}\left(S_{2}^{2}-C_{2}^{2}\right)]=0 \end{array}$$

where $C_{1}=\cos\beta_{1}L,\;C_{2}=\cos h\beta_{2}L,\;S_{1}=\sin\beta_{1}L,\;S_{2}=\sin h\beta_{2}L.$

Equation (46) is a transcendental equation with respect to the eigenvalues $\beta_{1},\;\;\beta_{2}$. The analytical solutions of $\beta_{1},\;\beta_{2}$ cannot be written exactly by conventional methods, but it is possible to obtain a series of values of λ by the numerical method. Then, the natural frequency in the OCI condition $f_{\mathrm{oz}}$ could be obtained. Under the short-circuit electrical condition, the surface induced electric potential of the flexoelectric energy harvester is zero. Hence, the natural frequency $f_{\mathrm{sz}}$ of the beam structure can be obtained by the same method of setting $\phi \left(t\right)=0$ in the corresponding boundary condition.

## 4. Numerical Analysis and Discussion

In the present study, the following dimensionless parameters are used [25]:

(47) $$K_{w}=k_{w}\frac{L^{4}}{D_{11}},\;K_{p}=k_{p}\frac{L^{2}}{D_{11}},\;{\tilde{H}}_{x}=H_{x}\sqrt{\frac{\xi bhL^{2}}{c_{11}I}},\;D_{11}\approx \frac{c_{11}h^{3}b}{12},\;I=\frac{bh^{3}}{12}.$$

### 4.1. Analysis of Static Bending Behavior of Flexoelectric Beam Structures

BaTiO_3_ has a large flexoelectric coefficient and significant electromechanical coupling properties. Therefore, as analyzing the bending behavior of the beam structure, BaTiO_3_ material is taken as the research object. The values of the selected parameters are [8,25,33,34]: elasticity coefficient $c_{11}=167.55\;\mathrm{GPa}$, piezoelectric coefficient $e_{311}=-4.4\;C/m^{2}$, dielectric constant $a_{33}=12.56\;\mathrm{nC}/\left(V\cdot m\right)$, electric polarization rate $\chi_{33}=12.46\;\mathrm{nC}/\left(V\cdot m\right)$, flexoelectric coefficient of about $10^{-5}-10^{-8}\;C/m$, L=50h, and b=h. In addition, the relationship between external force *F* and the beam thickness is taken to be $\left|F/h\right|=1\;N/m$. The dimensionless shear parameter $K_{P}=5$, the dimensionless linear parameter $K_{w}=5$, and the dimensionless magnetic field strength ${\tilde{H}}_{x}$=1 [25]. We define the expression of the normalized effective stiffness as $\frac{\int_{V}^{}\varepsilon_{e}c_{1111}\varepsilon_{e}dV}{\int_{V}^{}\varepsilon_{f}c_{1111}\varepsilon_{f}dV}$ [8,35], where $\varepsilon_{e}$ and $\varepsilon_{f}$ are the strain $\varepsilon_{11}$ in the beam structure without induced electric potential and with induced electric potential, respectively.

**Figure 2:**
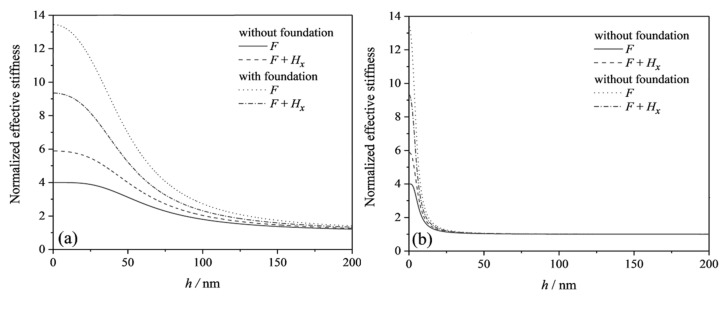
The normalized effective stiffness of flexoelectric cantilever beams with and without linear elastic substrate subjected to different applied loads under the OCI condition: (**a**) $\mu_{3113}=1\;\mu C/m$; (**b**) $\mu_{3113}=0.1\;\mu C/m$.

Figure 2 gives the curves of the normalized effective stiffness varying with beam thickness for two beam structures with different flexoelectric coefficients ($\mu_{3113}=0.1\;\mu C/m,\;1\;\mu C/m$). The figure shows that the effective stiffness increases with decreasing beam thickness. It indicates that the decrease in the size of the structure increases the flexoelectric effect, which in turn significantly affects the bending deformation of the beam. As the beam thickness decreases to a few nanometers, a saturation value of the effective stiffness gradually appears. Under different external conditions, the saturation values of the effective stiffness are different. This is because the reduction of the beam thickness under external loads causes the induced electric potential, which is generated by the flexoelectric effect, to increase firstly and then decrease. When the induced electric potential decreases, the reverse moment generated by the induced electric potential will also decrease. Therefore, the effective stiffness does not tend to infinity but reaches saturation values. The effective stiffness of the beam based on the linear elastic substrate is greater than that of a beam without substrate. This is explained as the beam structure and the substrate can deform together and can withstand relatively large deformations. The linear elasticity considers the normal pressure and the transverse shear stress from the surrounding elastic medium, which acts as a restraint on the deformation of the beam structure. The effective stiffness of the beam without the substrate is greater when both the force *F* and magnetic field are applied than when only force *F* is applied. FWhereas the effective stiffness of the flexoelectric beam structure based on the linear elastic substrate is smaller when both the force *F* and magnetic field are applied than when only force *F* is applied. Comparing Figure 2a,b, it can be found that the flexoelectric coefficient does not change saturation values of the effective stiffness. When the flexoelectric coefficient decreases to 0.1 μC/m, the critical thickness at which the effective stiffness reaches the saturation value decreases. This is because the decrease of the flexoelectric coefficient causes a significant decrease in the flexoelectric effect, when a beam with a smaller thickness is able to generate a larger strain gradient and produce a higher induced electric potential to bring the beam stiffness to reach its saturation value.

**Figure 3:**
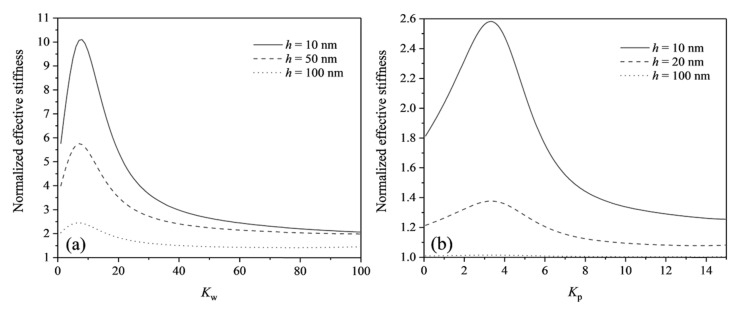
The normalized effective stiffness of flexoelectric cantilever beams with different thicknesses as a function of linear elastic parameters: (**a**) with linear parameters; (**b**) with shear parameters.

Figure 3a,b plots the curves of normalized effective stiffness of flexoelectric cantilever beams with different thicknesses as a function of linear or shear parameter. It can be observed from the figure that with the increase of linear and shear parameters, the effective stiffness first increases rapidly, and reaches a maximum value at about $K_{w}=8$ or $K_{p}=4$, then gradually decreases and stabilizes. The results show that the ability of the beam to resist bending deformation first increases and then decreases with the increase of the linear and shear parameters. In addition, the smaller the beam thickness, the more significant the effect of the linear elastic parameters. Therefore, when people design or select flexoelectric devices based on linear elastic substrates at the nano-scale, selecting reasonable substrate parameters can make the devices obtain optimal mechanical properties or electrical properties.

**Figure 4:**
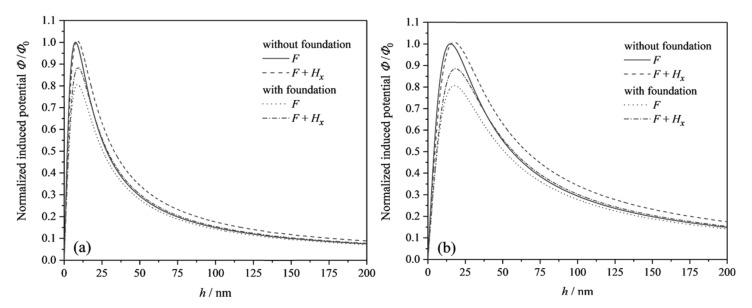
The normalized induced electric potential for flexoelectric cantilever beams with and without linear elastic substrates under the OCI condition: (**a**) $\mu_{3113}=0.1\;\mu C/m$; (**b**) $\mu_{3113}=0.2\;\mu C/m$.

We define the normalized induced electric potential as $\phi_{i}/\phi_{0i}$ (*i* = 1, 2, 3, 4, $\phi_{0i}$ denotes the maximum induced potential of the beam without substrates subjected to force *F* only). Figure 4a,b plots the induced electric potential as a function of thicknesses for two cantilever beam structures with different flexoelectric coefficients ($\mu_{3113}=0.1\;\mu C/m$, 0.2 μC/m). The induced electric potential of the flexoelectric beam structure increases and then decreases gradually to a stable value with increasing the beam thickness. The peak induced electric potential of the flexoelectric beam based on the linear elastic substrate is smaller than that of the beam without the substrate. The linear elastic substrate acts as a mechanical boundary effect. The restraint on the beam will reduce the bending deformation of the beam, which in turn reduces the induced electric potential generated by the bending deformation. The curves of the normalized induced electric potential with the magnetic field are shifted upward to the right with respect to the beam structure with *F* only. In this case, the magnetoelectric effect of the beam structure causes a change in the electrical properties. It can be recognized that a large strain gradient is not necessary to generate the induced electric potential when a magnetic field is present. In addition, the comparison between Figure 4a,b shows that the beam structure with a large flexoelectric coefficient has a larger beam thickness corresponding to the peak induced electric potential. These conclusions can be used to design flexoelectric nanostructures and flexoelectric materials with optimal dimensions to obtain the best electrical output.

### 4.2. Analysis of The Vibration Characteristics of Flexoelectric Beam Structures

BaTiO_3_ has a high stiffness and a very high natural frequency, which are not suitable for practical application. It is possible to choose a less stiff material as the base material to reduce the natural frequency of flexoelectric beam structures at the nano-scale. Therefore, in the analysis of the vibration characteristics of the flexoelectric cantilever beam, polyvinylidene fluoride (PVDF) is used. The parameters of PVDF are as follows [36]: piezoelectric coefficient $e_{311}=-0.01\;N/\left(V\cdot m\right)$, dielectric coefficient $a_{33}=8.15\times 10^{-11}\;C^{2}/\left(N\cdot m^{2}\right)$, elasticity coefficient $c_{11}=3.7\;\mathrm{GPa}$, and density $\rho =1.78\times 10^{3}\;\mathrm{kg}/m^{3}$. The flexoelectric coefficient is taken to be about $10^{-6}\sim 10^{-9}\;C/m$ [17,35]. The length, width, and thickness ratios are kept at 100: 10: 1. K is used to represent the proportion of the mass of the end mass block in the entire flexoelectric beam structure, and the expression of the end mass block is $M_{t}=m\times L\times K$, where K is taken as 0.05.

**Figure 5:**
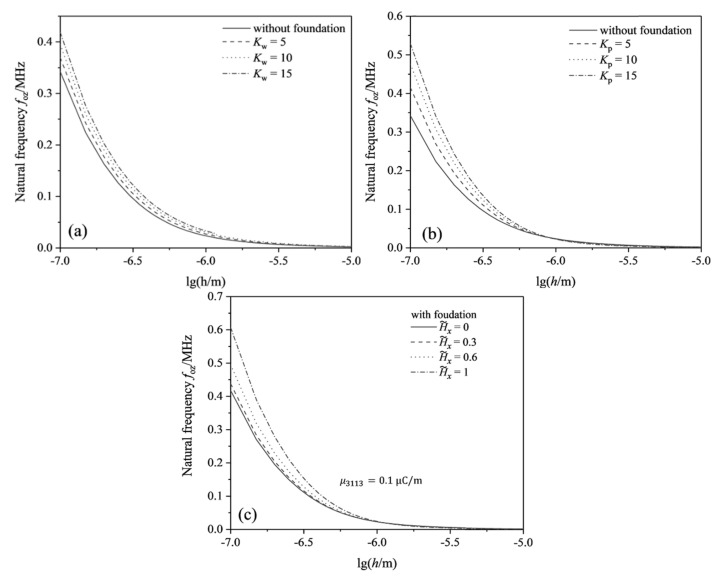
The variation of natural frequency of flexoelectric cantilever beams as beam thickness under the OCI condition: (**a**) with different linear parameters, $K_{p}=0$, ${\tilde{H}}_{x}=0$; (**b**) with different shear parameters, $K_{w}=0$, ${\tilde{H}}_{x}=0$; (**c**) with different magnetic field,$\;K_{p}=5$, $K_{w}=5$.

Figure 5 illustrates the natural frequency of the flexoelectric beam based on the linear elastic substrate with the beam thickness under different linear parameters, shear parameters, and the magnetic field strength. The figure indicates that the natural frequency of the cantilever beam with and without the substrate decreases with increasing the thickness, which is the same as that of Lin et al. [17]. Figure 5a,b shows that the natural frequency of the cantilever beam structure with the same thickness will increase with the increase of the linear parameters or shear parameters, and the shear parameters have a greater influence on the natural frequency. The increase of the linear elastic parameters can significantly increase the stiffness of the nanobeam, which has a similar effect as the flexoelectric coefficient, and finally increases the natural frequency of the beam structure. Figure 5c shows that when the magnetic field strength increases, the natural frequency also increases. The magnetic field acts similarly as the shearing effect of the substrate, which reduces the bending deformation and greatly increases the natural frequency of the flexoelectric beam.

**Figure 6:**
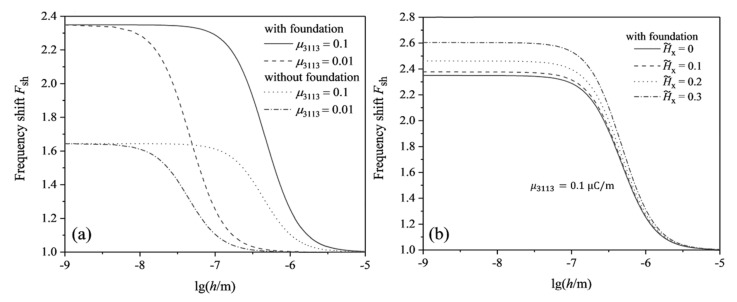
The effective frequency shift of flexoelectric cantilever beams under the OCI condition: (**a**) with and without linear elastic substrates,$\;{\tilde{H}}_{x}=0$, $\mu_{3113}=0.1\;\mu C/m$, $\mu_{3113}=0.01\;\mu C/m$; (**b**) with different magnetic fields,$\;K_{p}=5$,$\;K_{w}=5$,$\;\mu_{3113}=0.1\;\mu C/m$.

The natural frequency shift of the beam structure is an important parameter for judging the electromechanical coupling performance and vibration characteristics of the flexoelectric harvesters [31,32,37]. In the present paper, the effective frequency shift of the flexoelectric cantilever beam structure is defined as $F_{\mathrm{sh}}=f_{\mathrm{oz}}/f_{\mathrm{sz}}$. Figure 6a is presented to investigate the effective frequency shift of the flexoelectric beam with and without the linear elastic substrate as a function of beam thickness for two different flexoelectric coefficients (0.1 μC/m, 0.01 μC/m). The results show that the effective frequency shift of the flexoelectric beam based on the linear elastic substrate is higher than that of the beam without the substrate. The effective frequency shift saturation value is the same for the identical flexoelectric beam structure with different flexoelectric coefficients. The critical thickness corresponding to the saturation of the effective frequency shift decreases gradually when the flexoelectric coefficient decreases. Figure 6b gives the curves of the effective frequency shift with the thickness of flexoelectric beam structures based on a linear elastic substrate under different magnetic fields. It can be seen from the Figure 6b that the effective frequency shift of the cantilever beam structure increases as the applied magnetic field strength increases. The result indicates that the addition of the magnetic field improves the ability of the beam to resist bending deformation and significantly affects the natural frequency of the flexoelectric beam structure under the OCI condition, which in turn changes the effective frequency shift of the structure.

## 5. Conclusions

In this paper, the static bending behavior and vibration characteristics of flexoelectric cantilever beams attached on linear elastic substrates are studied and analyzed. Based on the electrical Gibbs free energy function, the dynamic governing equation and the corresponding electromechanical boundary conditions are obtained using the Hamilton’s variational principle. For the static problem, the deflection and the induced electric potential have been solved and expressed analytically. Further, the characteristic equations of the natural frequency of beam structures are derived, and thus the dynamic characteristics of the structures are analyzed. The numerical results show that both the applied magnetic field and the linear elastic substrate significantly improve the resistance of flexoelectric beams to bending deformation. The beam can achieve an optimal mechanical performance as $K_{w}=8$ or $K_{p}=4$. The peak induced electric potential of the flexoelectric beam based on the linear elastic substrate will be lower than that of the beam without the substrate. The increase of the flexoelectric coefficient, linear elastic parameters and the magnetic field strength will increase the natural frequency of the beam structure. As the thickness of the flexoelectric beam decreases, the effective frequency shift gradually increases to a saturation value, which is related to the end mass block, the linear elastic parameters, and the magnetic field strength. The critical beam thickness for reaching the saturation value is related to the flexoelectric coefficient only.
